# Preparation and Evaluation of Novel Folate Isonitrile ^99m^Tc Complexes as Potential Tumor Imaging Agents to Target Folate Receptors

**DOI:** 10.3390/molecules26154552

**Published:** 2021-07-28

**Authors:** Junhong Feng, Xuran Zhang, Qing Ruan, Yuhao Jiang, Junbo Zhang

**Affiliations:** 1 Key Laboratory of Radiopharmaceuticals of Ministry of Education, College of Chemistry, Beijing Normal University, Beijing 100875, China; 201921150102@mail.bnu.edu.cn (J.F.); 201321150179@mail.bnu.edu.cn (X.Z.); 201931150055@mail.bnu.edu.cn (Q.R.); 201921150103@mail.bnu.edu.cn (Y.J.); 2 NMPA Key Laboratory for Research and Evaluation of Radiopharmaceuticals (National Medical Products Administration), Perking University, Beijing 100091, China

**Keywords:** ^99m^Tc, isonitrile, folate receptor, SPECT/CT imaging

## Abstract

In order to seek novel technetium-99m folate receptor-targeting agents, two folate derivatives (CN5FA and CNPFA) were synthesized and radiolabeled to obtain [^99m^Tc]Tc-CN5FA and [^99m^Tc]Tc-CNPFA complexes, which exhibited high radiochemical purity (>95%) without purification, hydrophilicity, and good stability in vitro. The KB cell competitive binding experiments indicated that [^99m^Tc]Tc-CN5FA and [^99m^Tc]Tc-CNPFA had specificity to folate receptor. Biodistribution studies in KB tumor-bearing mice illustrated that [^99m^Tc]Tc-CN5FA and [^99m^Tc]Tc-CNPFA had specific tumor uptake. Compared with [^99m^Tc]Tc-CN5FA, the tumor/muscle ratios of [^99m^Tc]Tc-CNPFA were higher, resulting in a better SPECT/CT imaging background. According to the results, the two ^99m^Tc complexes have potential as tumor imaging agents to target folate receptors.

## 1. Introduction

Tumors are pathological changes caused by the uncontrolled and continuous proliferation of cells under the influence of various factors [1]. Folate receptor (FR) is a 38 kDa glycoprotein linked to the cell membrane by glycan phosphatidylinositol. It is overexpressed on the surface of the cell membrane in the majority of human tumors. FR mainly consists of α, β, and γ subtypes [2]; in particular, folate α-receptors are highly conserved in healthy cells but highly expressed in many epithelial-derived malignancies, such as ovarian, breast, endometrial, lung, and nasopharyngeal cancer cells. Thus, the α-subtype of the folate receptor has become a hotspot of radiation-targeted drug research for the diagnosis and therapy of high-expression tumors [3,4].

In recent years, nuclear medicine imaging has shown the advantages of being sensitive, specific, noninvasive, and able to detect changes in molecular biological behavior and pathophysiology in vivo. It has become one of the most important diagnostic methods for cancer. With the widespread use of single-photon emission computed tomography (SPECT) and positron emission tomography (PET), radionuclide tumor imaging has become one of the advantages of nuclear medicine [5,6,7].

^99m^Tc is the most widely used SPECT radionuclide with good nuclide properties, including a half-life of 6.02 h, emission of 140 keV γ-rays, and the advantages of coordinated chemical diversity. Therefore, ^99m^Tc complexes are often used in the clinical diagnosis of some diseases, accounting for more than 85% of nuclear medicine SPECT images [8,9]. Recently, a number of ^99m^Tc-labeled folate-based conjugates have been developed as potential folate receptor imaging agents [10,11,12,13,14]. Although these complexes have shown high tumor uptake, most of the reported radiopharmaceuticals targeting folate receptor require purification by high-performance liquid chromatography (HPLC), thus limiting their application in clinical practice. Therefore, developing novel ^99m^Tc-labeled folate-based conjugates for folate receptor imaging with no further purification is worthwhile.

For the design of bifunctional radiopharmaceuticals, there are four important components: targeting group, linker, bifunctional chelator (BFC), and radionuclide [15]. Isocyanate (–NC) is a strong coordination group that can form a stable ^99m^Tc complex with six coordination sites. Because the ^99m^Tc-labeled isocyanate-based complex had six isocyanate ligands which contain targeting group, its targeting ability to the target could be enhanced [16,17,18]. Recently, preclinical studies of ^99m^Tc-CN5DG and ^99m^Tc-CN7DG demonstrated that they could be promising candidates for tumor imaging [19,20]. The introduction of an ethylene glycol (PEG) chain can increase the hydrophilicity of the drug, thus improving its metabolic pathway in vivo [21,22,23].

Considering the evidence above, we aimed to develop novel ^99m^Tc-labeled folate-based conjugates as tumor imaging agents. We synthesized two folate-based conjugates with an isonitrile group (CN5FA and CNPFA) and labeled them with ^99m^Tc to produce [^99m^Tc]Tc-CN5FA and [^99m^Tc]Tc-CNPFA. Their potential for tumor imaging was evaluated for the first time.

## 2. Results

### 2.1. Synthesis

**Scheme 1:**
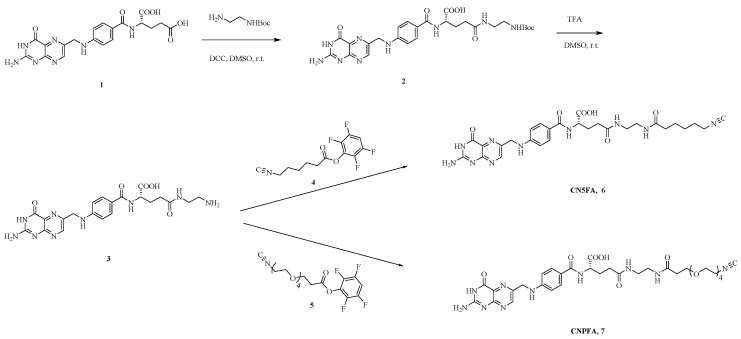
Synthesis of CN5FA and CNPFA.

The reaction route is shown in Scheme 1.

### 2.2. Radiolabeling

**Scheme 2:**
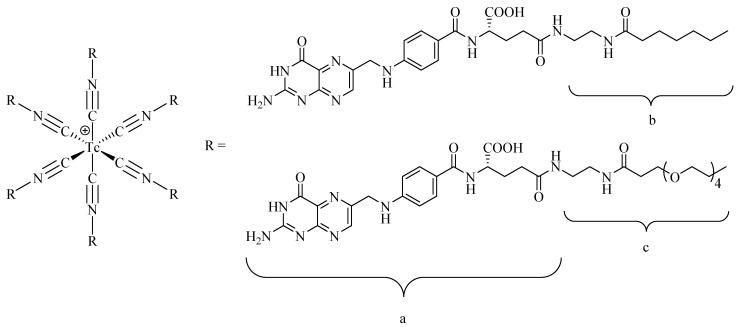
The proposed structures of the radiolabeled tracers of [^99m^Tc]Tc-CN5FA and [^99m^Tc]Tc-CNPFA (“a” stands for target group, “b” and “c” stand for linkers, “–CN” stands for BFC, technetium-99m as a nuclide).

**Figure 1:**
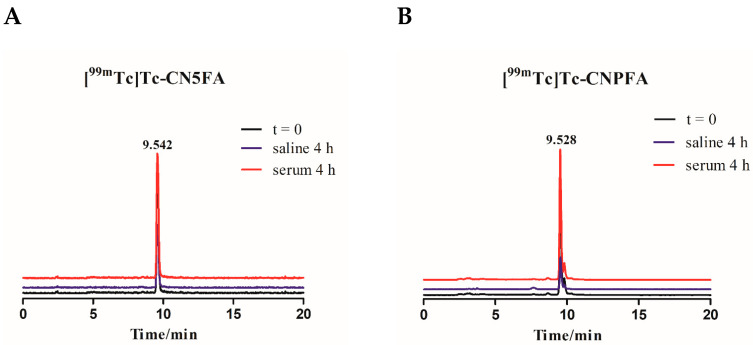
Radiochromatograms of [^99m^Tc]Tc-CN5FA (**A**) and [^99m^Tc]Tc-CNPFA (**B**) in original labeling solution without purification (black line), as well as with incubation in saline at room temperature for 4 h (blue line) and in mouse serum at 37 °C for 4 h (red line).

[^99m^Tc]Tc-CN5FA and [^99m^Tc]Tc-CNPFA (the proposed structures of the radiolabeled tracers are shown in Scheme 2) were easily prepared in high radiochemical purity (RCP > 95%) without purification using a developed kit formulation in 20 min at 100 °C. The resulting solutions were analyzed by instant thin-layer chromatography silica gel strips (ITLC/SG) and high-performance liquid chromatography (HPLC). The ITLC/SG results of [^99m^Tc]Tc-CN5FA and [^99m^Tc]Tc-CNPFA were as follows: in anticoagulant acid citrate dextrose (ACD) solution, [^99m^Tc]Tc-CN5FA and [^99m^Tc]Tc-CNPFA stayed at the origin (R_f_ = 0–0.1), while ^99m^TcO_4_^−^ (R_f_ = 0.9–1.0) and ^99m^TcO_2_·nH_2_O (R_f_ = 0.8–1.0) were moved to the front. The HPLC results of [^99m^Tc]Tc-CN5FA and [^99m^Tc]Tc-CNPFA are shown in Figure 1. The molar activities (Am) of [^99m^Tc]Tc-CN5FA and [^99m^Tc]Tc-CNPFA were about 2.24–22.46 GBq/µmol and 2.74–27.42 GBq/µmol, respectively.

### 2.3. Physicochemical Properties Evaluation

#### 2.3.1. Stability Study

As shown in Figure 1, after incubation in the radiolabeling solution at room temperature for 4 h or in serum at 37 °C for 4 h, the RCPs of the complexes remained at more than 95%. Nearly no decomposition of [^99m^Tc]Tc-CN5FA and [^99m^Tc]Tc-CNPFA was found, indicating their good stability in vitro.

#### 2.3.2. Partition Coefficient 

**Figure 2:**
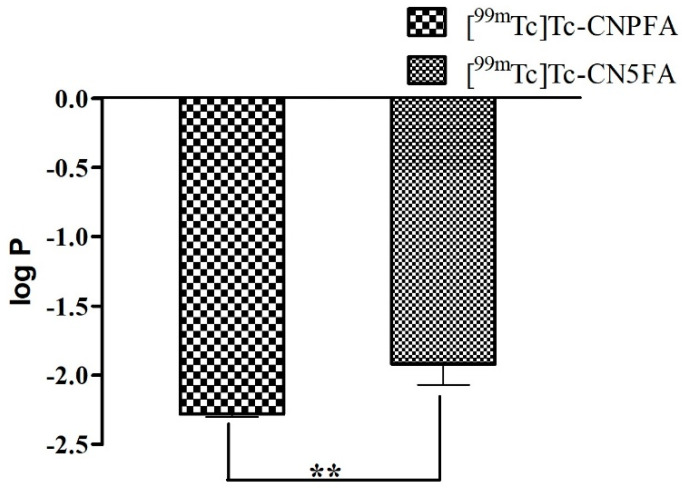
The log P of [^99m^Tc]Tc-CN5FA and [^99m^Tc]Tc-CNPFA (** *p* < 0.01).

The partition coefficient values, log P (P = the radioactivity of the organic phase/the radioactivity of the water phase) of [^99m^Tc]Tc-CN5FA and [^99m^Tc]Tc-CNPFA were −1.92 ± 0.15 and −2.28 ± 0.02, respectively. This result suggested that both complexes were hydrophilic and that [^99m^Tc]Tc-CNPFA had a better hydrophilicity than [^99m^Tc]Tc-CN5FA due to the ethylene glycol chain linker (Figure 2).

### 2.4. In Vitro Binding with KB Cells

**Figure 3:**
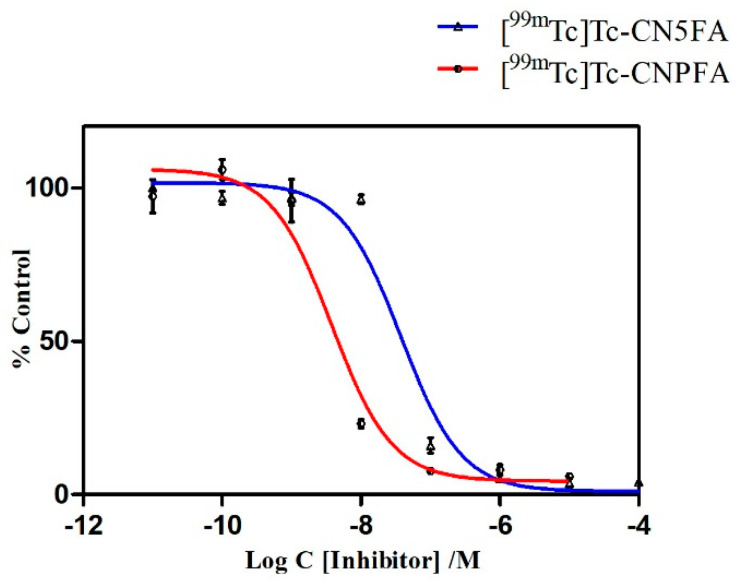
In vitro competitive binding curves of [^99m^Tc]Tc-CN5FA and [^99m^Tc]Tc-CNPFA.

To confirm the FR binding property of acquired ^99m^Tc-labeled complexes, in vitro competitive binding experiments were designed with KB cells. In vitro competitive binding curves of [^99m^Tc]Tc-CN5FA and [^99m^Tc]Tc-CNPFA are shown in Figure 3. These findings imply that the IC_50_ values of [^99m^Tc]Tc-CN5FA and [^99m^Tc]Tc-CNPFA were 37.92 ± 0.41 nM and 3.80 ± 0.07 nM with KB cells, respectively. This result suggests that both complexes specifically bound to the folate receptor.

### 2.5. Biodistribution

**Figure 4:**
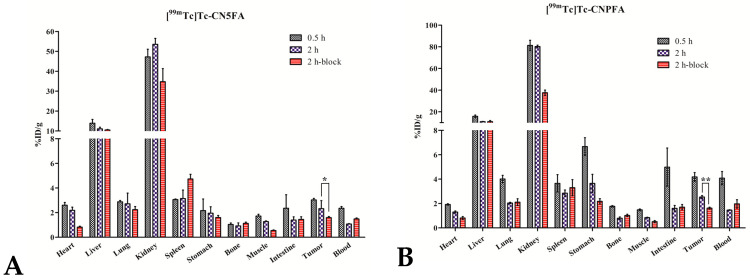

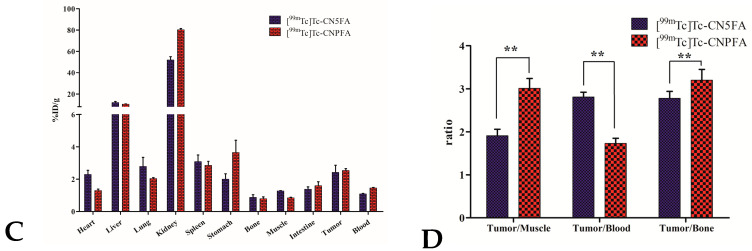
(**A**) Biodistribution of [^99m^Tc]Tc-CN5FA in KB tumor-bearing mice (* *p* < 0.05); (**B**) biodistribution of [^99m^Tc]Tc-CNPFA in KB tumor-bearing mice (** *p* < 0.01); (**C**) biodistribution of [^99m^Tc]Tc-CN5FA and [^99m^Tc]Tc-CNPFA in KB tumor-bearing mice at 2 h p.i.; (**D**) the ratios of tumor-to-nontargeted tissues of [^99m^Tc]Tc-CN5FA and [^99m^Tc]Tc-CNPFA.

The biological distribution results in KB tumor-bearing mice are shown in Table 1. The results showed that the tumor uptakes of [^99m^Tc]Tc-CN5FA and [^99m^Tc]Tc-CNPFA were 2.42 ± 0.44 %ID/g and 2.53 ± 0.12 %ID/g at 2 h post injection (p.i.), respectively, which were significantly reduced to 1.53 ± 0.10 %ID/g and 1.62 ± 0.08 %ID/g at 2 h p.i. by pre-administration of free folate acid (FA) (about 4 × 10^4^ µg/kg). This indicates that they were specific to the folate receptor. As the results demonstrate, the tumor/muscle ratios of [^99m^Tc]Tc-CNPFA were 2.82 at 0.5 h p.i. and 3.01 at 2 h p.i., which were better than 1.76 at 0.5 h p.i. and 1.91 at 2 h p.i. for [^99m^Tc]Tc-CN5FA (Figure 4).

For other organs, the radioactive uptake in the kidney is relatively high due to the high FR expression in kidney proximal tubule cells. In addition, the radioactive concentrations in the liver and intestines are also appreciable.

### 2.6. SPECT/CT Imaging Study

**Figure 5:**
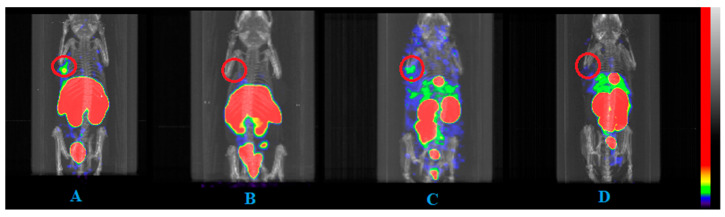
(**A**) SPECT/CT images of [^99m^Tc]Tc-CN5FA in KB tumor-bearing mice and (**B**) the blocking study at 2 h p.i.; (**C**) SPECT/CT images of [^99m^Tc]Tc-CNPFA in KB tumor-bearing mice and (**D**) the blocking study at 2 h p.i.

SPECT/CT imaging studies of [^99m^Tc]Tc-CN5FA and [^99m^Tc]Tc-CNPFA in KB tumor-bearing mice are shown in Figure 5. The SPECT/CT imaging results were consistent with the biodistribution results. The tumor lesions were clearly visualized by both probes at 2 h post injection. Kidney and tumor uptake significantly decreased in the blocking experiments by pre-administration of excess FA (about 4 × 10^4^ µg/kg). Interestingly, the imaging effect of [^99m^Tc]Tc-CNPFA was superior to that of [^99m^Tc]Tc-CN5FA because the former had a higher T/NT ratio than the latter.

## 3. Discussion

In recent years, receptor-mediated targeted molecular probes have become the focus of research and development of radiopharmaceuticals. Compared with other targeted transport systems, the folate molecule has the advantages of strong affinity, small molecular weight, low cost, easy structural modification, good chemical and biological stability, and no immunogenicity. Folate receptor drugs have become a hot topic of targeted drug research. Folate and its analogues were coupled with a radionuclide to form tracers, which can specifically bind to FR and selectively concentrate in tissues rich in FR expression. Because of the significant difference in the distribution of FR in tumor and nontumor tissues, FR-mediated tracers can obtain high-contrast images of tumor and normal tissues to diagnose, locate, and evaluate the effect of chemotherapy [24,25]. At present, ^66/67/68^Ga, ^111^In, ^99m^Tc, and ^18^F have been reported as radionuclides for preparing targeted folate receptor radiopharmaceuticals [26,27,28,29]. 

At present, most studies on folate-mediated radiopharmaceuticals are limited to the preclinical studies, and most folate imaging agents need to be separated and purified by HPLC. To promote clinical application, finding a better labeling method and better linker, simplifying the preparation process, and improving drug targeting in vivo are the problems to be solved in the future.

In this work, we used folate as a target molecule, isonitrile as a BFC, methylene chain and PEG chain as linkers, and ^99m^Tc as a radionuclide to discover novel tracers for folate receptor imaging. Two ^99m^Tc-labeled complexes ([^99m^Tc]Tc-CN5FA and [^99m^Tc]Tc-CNPFA) were successfully prepared, and the RCP of [^99m^Tc]Tc-CN5FA and [^99m^Tc]Tc-CNPFA was over 95% without purification. 

In the biodistribution experiment stage, because of the poor solubility of ligand CN5FA, the biodistribution results showed obvious concentrations in the liver (52.35 ± 1.19 %ID/g at 2 h p.i.) and other abdominal organs when the labeled condition was under weak acid. Therefore, to reduce the concentration of radioactivity in the liver and other abdominal organs, the pH of the labeling solution was changed to weakly alkaline in the follow-up experiments so that the ligand CN5FA could be better dissolved.

As shown in Table 1, the tumor uptake of [^99m^Tc]Tc-CN5FA was 3.03 ± 0.10 %ID/g and that of [^99m^Tc]Tc-CNPFA was 4.18 ± 0.36 %ID/g at 0.5 h p.i., suggesting that [^99m^Tc]Tc-CNPFA had a higher tumor uptake. However, there was no significant difference in tumor uptake between the two complexes at 2 h p.i. The muscle uptake of [^99m^Tc]Tc-CNPFA (0.84 ± 0.04 %ID/g) was lower than that of [^99m^Tc]Tc-CN5FA (1.27 ± 0.03 %ID/g), thus leading to a higher tumor/muscle ratio of the former than that of the latter. In the inhibition experiments, with early injection of excessive FA, the tumor and kidney uptake of both complexes was shown to have an inhibitory effect of more than one-third at 2 h p.i.

In 2019, Lodhi et al. developed a folate receptor tracer [^99m^Tc]Tc-10 using isonitrile (–NC) as the BFC [14]. In contrast to our work, the results showed that [^99m^Tc]Tc-10 had higher tumor uptake (4.02 ± 0.80 %ID/g) and lower liver concentration (5.14 ± 1.79 %ID/g) at 2 h post injection. However, the preparation of [^99m^Tc]Tc-10 was a two-step process in which the [^99m^Tc(H_2_O)_3_(CO)_3_]^+^ intermediate needs to be prepared first before the product required to be purified by HPLC. Compared with the method of direct labeling of [^99m^Tc]Tc-CN5FA and [^99m^Tc]Tc-CNPFA, the preparation process of [^99m^Tc]Tc-10 was tedious, time-consuming, and not suitable for clinical application.

As shown in Figure 5, SPECT/CT results showed that both complexes had significant radioactivity concentrations in tumor and kidney sites, and radioactivity concentrations in the liver and intestinal tract were also observed, suggesting that, in addition to urine metabolism, the liver and intestine may also be involved in metabolism to a certain extent. At the same time, inhibition assays demonstrated that these two probes could bind specifically to KB tumor with high FR expression.

## 4. Materials and Methods

All chemical reagents and solvents were purchased from commercial sources and were used without further purification. Blank kits were acquired from Beijing Shihong Pharmaceutical Center, Beijing Normal University, China. The ^99^Mo/^99m^Tc generator was obtained from Atomic High Tech Co. Ltd., China. NMR spectra were recorded on a 400 MHz spectrophotometer (JEOL, Tokyo, Japan). HRMS spectra were recorded on a Bruker microTOF-QII mass spectrometer and a Bruker solariX ESI mass spectrometer. HPLC was acquired from a SHIMADZU system (CL-20AVP) equipped with an SPD-20A UV detector (λ = 254 nm) and a Bioscan flflow count 3200 NaI/PMT γ-radiation scintillation detector. The SPECT/CT imaging studies were obtained with a Triumph SPECT/CT scanner (TriFoil Imaging, Los Angeles, CA, USA). The human nasopharyngeal KB carcinoma cell line was purchased from the National Laboratory Cell Resource Sharing Service Platform (Beijing, China). BALB/c nude mice were purchased from Beijing Vital River Laboratory Animal Technology Co., Ltd. (Beijing, China). Animal studies were performed according to the Regulation on Laboratory Animals of Beijing Municipality and guidelines of the Ethics Committee of Beijing Normal University (permit no. CLS-EAW-2018-001). 

### 4.1. Synthesis of CN5FA and CNPFA

*N^2^-(4-(((2-Amino-4-oxo-3,4-dihydropteridin-6-yl)methyl)amino)benzoyl)-N^5^-(2-aminoethyl)-l-glutamine* (**3**): The precursor *N*-(2-aminoethyl)-folate acid was prepared according to the previously reported method [14]. Under nitrogen atmosphere, *N*-Boc-ethylenediamine (247 mg, 1.5 mmol) and DCC (536 mg, 2.6 mmol) were added to the DMSO solution containing FA (662 mg, 1.5 mmol, **1**). The reaction took place overnight at room temperature and in the dark. The reaction liquid was filtered to remove the residue, and the filtrate was collected. The yellow solid product **2** was obtained by repeatedly washing the filtrate with cold ether. ^1^H-NMR (400 MHz, DMSO-*d*_6_) δ 8.61 (s, 1H), 7.61 (m, 2H), 6.60 (m, 2H), 4.44 (s, 2H), 4.33–4.20 (m, 1H), 3.05–3.02 (m, 2H), 2.93–2.88 (m, 2H), 2.33–1.94 (m, 2H), 1.83–1.53 (m, 2H), 9.31 (m, 9H).

The TFA was added to the DMSO solution containing compound **2** and reacted at room temperature for 3 h. After removing TFA in vacuo, the yellow product **3** was obtained by washing with cold ether several times (458 mg, 63.2%). ^1^H-NMR (400 MHz, DMSO-*d*_6_) δ 8.62–8.60 (m, 1H), 7.62 (m, 2H), 6.63–6.57 (m, 2H), 4.45 (s, 2H), 4.32–4.24 (m, 1H), 3.29–3.21 (m, 2H), 2.82–2.76 (m, 2H), 1.91–1.73 (m, 2H), 1.71–1.56 (m, 2H).

2,3,5,6-Tetrafluorophenyl 6-isocyanohexanoate (**4**) and 2,3,5,6-tetrafluorophenyl 3-(2-isocyanoethoxy)propanoate (**5**) were synthesized according to the literature [20]. 

*N^2^-(4-(((2-Amino-4-oxo-3,4-dihydropteridin-6-yl)methyl)amino)benzoyl)-N^5^-(2-(6-isocyanohexanamido)ethyl)-l-glutamine* (*CN5FA,* **6***)*: Compound **4** (411 mg, 0.8 mmol) and triethylamine (TEA) were added to the DMSO solution containing compound **3** (287 mg, 1.0 mmol) under nitrogen atmosphere. The reaction was stirred violently for 5 h at room temperature and in the dark. The yellow solid product **6** was obtained by repeatedly washing the filtrate with cold ether (225 mg, 46.3%). ^1^H-NMR (400 MHz, DMSO-*d*_6_) δ 8.60 (s, 1H), 7.61 (s, 2H), 6.59 (s, 2H), 4.44 (s, 2H), 4.28–4.17 (m, 1H), 3.04 (s, 4H), 2.92 (d, *J* = 6.4 Hz, 2H), 1.99 (d, *J* = 6.1 Hz, 2H), 1.49 (s, 4H), 1.43 (d, *J* = 7.0 Hz, 2H), 1.24 (d, *J* = 6.4 Hz, 2H), 1.07 (d, *J* = 6.5 Hz, 2H). ^13^C-NMR (100 MHz, DMSO-*d*_6_) δ 174.42, 171.89, 166.09, 161.25, 160.81, 156.27, 155.41, 153.97, 153.35, 150.65, 148.42, 129.08, 127.92, 111.07, 52.58, 45.98, 40.96, 38.32, 36.96, 35.14, 28.83, 28.03, 26.02, 25.37, 24.26. IR(KBr)/cm^−1^: 2148.79 (–NC). HRMS (*m*/*z*): found 607.2738, calculated 607.2735.

*N^2^-(4-(((2-amino-4-oxo-3,4-dihydropteridin-6-yl)methyl)amino)benzoyl)-N^5^-(2-(3-(2-isocyanoethoxy)propanamido)ethyl)-l-glutamine (CNPFA,***7***)*: Similar to the synthesis of compound **6**, Compound **5** (97 mg, 0.2 mmol) and TEA were added to a DMSO solution containing compound **3** (127 mg, 0.3 mmol) under nitrogen atmosphere. The reaction was stirred violently for 5 h at room temperature and in the dark. The yellow solid product **7** was obtained by repeatedly washing the filtrate with cold ether (75 mg, 50.7%). ^1^H-NMR (400 MHz, D_2_O) δ 8.71 (s, 1H), 7.84–7.72 (m, 2H), 6.97–6.85 (m, 2H), 4.67 (s, 2H), 4.48–4.35 (m, 1H), 3.72–3.61 (m, 12H), 3.42 (m, 6H), 3.33–3.20 (m, 4H), 2.51–2.33 (m, 6H). ^13^C-NMR (100 MHz, DMSO-*d*_6_) δ 173.99, 171.80, 170.10, 166.04, 162.34, 161.16, 155.78, 153.78, 150.73, 148.61, 129.02, 127.84, 121.45, 111.17, 69.66, 69.51, 67.89, 66.68, 45.80, 41.64, 38.43, 37.20, 36.16, 35.78, 32.04, 31.48, 28.13. IR(KBr)/cm^−1^: 2152.65 (–NC). HRMS (m/z): found 741.3320, calculated 741.3314.

### 4.2. Radiolabeling of [^99m^Tc]Tc-CN5FA and [^99m^Tc]Tc-CNPFA and Quality Control Assay

The [^99m^Tc]Tc-CN5FA and [^99m^Tc]Tc-CNPFA complexes were obtained by adding saline to blank kits containing 0.06 mg of SnCl_2_·2H_2_O, 1 mg of sodium citrate, 1 mg of l-cysteine, 5 mg of mannitol, 10 µg of CN5FA (compound **6**) or CNPFA (compound **7**), and freshly washed Na^99m^TcO_4_ solution (37–370 MBq) in turn, adjusting the pH of the solution to approximately 8.0 with NaOH (0.1 M) and reacting for 20 min at 100 °C.

The complexes were determined by ITLC/SG of the ACD system and radio-HPLC. The ITLC/SG trip was cut into 10 equal parts, and radioactive counting of ITLC strips was determined by a γ-counter. ITLC results of both complexes were as follows: in ACD solution, [^99m^Tc]Tc-CN5FA and [^99m^Tc]Tc-CNPFA stayed at the origin (R_f_ = 0–0.1), while ^99m^TcO_4_^−^ (R_f_ = 0.9–1.0) and ^99m^TcO_2_·nH_2_O (R_f_ = 0.8–1.0) were moved to the front. Radio-HPLC was analyzed by a mobile phase system consisting of 0.1% TFA water (solvent A) and 0.1% TFA acetonitrile (solvent B) with a gradient of 0−2 min 10% B, 2−5 min 10%−90% B, 10−20 min 90% B, and 20−25 min 90%−10% B (system B) at a flow rate of 1 mL/min. All experiments were performed in triplicate.

### 4.3. Stability Study

The stability of [^99m^Tc]Tc-CN5FA and [^99m^Tc]Tc-CNPFA in vitro was evaluated by measuring the RCP of the complexes. Stability in saline was achieved by holding the solution at room temperature for 4 h. Stability in the serum was achieved by mixing 100 μL of [^99m^Tc]Tc-CN5FA or [^99m^Tc]Tc-CNPFA with 200 μL of mouse serum at 37 °C for 4 h. After precipitation using acetonitrile, centrifugation, and concentration, the solutions were analyzed by radio-HPLC.

### 4.4. Determination of Partition Coefficient

The partition coefficient was measured as follows: 1.9 mL of phosphate buffer solution (0.025 M, pH 7.4), 2.0 mL of 1-octanol, and 0.1 mL of ^99m^Tc-CN5FA or ^99m^Tc-CNPFA solution were added to the centrifuge tube, and the sample was swirled for 5 min and centrifuged for 5 min (3000 r/min). Then, the radioactivity counts of the two phases were measured by taking 0.1 mL from the organic phase and the water phase. The partition coefficient P was calculated. The final partition coefficient was expressed as log P ± SD.

### 4.5. In Vitro Binding with KB Cells

KB cells were cultured with FFRPMI 1640 medium 24 h in advance on a 48-well plate with 5 × 10^4^ cells per well. The labeling solution was diluted to 5 μCi/mL with the same medium after growing, and the inhibitor was prepared at different concentrations. The 48-well plates were divided into eight groups, and each group was incubated with 100 μL of inhibitor solution, 100 μL of diluted labeled solution (5 μCi/mL), and 200 μL of medium at 37 °C for 2 h.

After removing the cell plate from the incubator, the medium was removed, and the cells were washed with PBS (0.1 M, pH 7.4). The washed cells were lysed with NaOH (1 M) for 5 min. Pyrolysates were collected in the hoses, and the radioactivity count of each hose was measured by a γ-counter. The data were analyzed with GraphPad Prism 5.0 and nonlinearly fit to the log IC_50_ ± SD value.

### 4.6. Biodistribution Study in KB Tumor-Bearing Mice

The biodistribution experiments were divided into normal groups and inhibition groups (*n* = 3), in which the inhibition group was injected with inhibitor (about 4 × 10^4^ µg/kg) 30 min in advance. The KB tumor-bearing mice were decapitated 30 and 120 min after the tail vein injection of 0.1 mL labeled solution (about 7.4 × 10^4^ Bq). The heart, liver, lung, kidney, spleen, stomach, bone, muscle, intestine, tumor, blood, and other related tissues and organs were wiped clean and weighed, and the radioactivity count was measured. Each tissue per gram of injection dose (%ID/g) was calculated.

### 4.7. SPECT/CT Imaging Study in KB Tumor-Bearing Mice

The SPECT/CT imaging experiments were also divided into a normal group and an inhibition group (*n* = 3). The labeled solution (about 18.5 MBq) was injected into the tail vein of nude mice bearing KB tumor. The inhibition group was injected with 100 µg FA (about 4 × 10^4^ µg/kg) 30 min in advance. After 2 h, the SPECT/CT scan was performed in anesthetized mice by using 1.5% isoflurane. The SPECT acquisition (peak value 140 keV, 20% width, 90° rotation, MMP 919 Collimator) was performed after 4 min of CT scanning (512 views, 2 × 2 binding, 75 kV, exposure time 230 ms). SPECT/CT images were made using HiSPECT software and vivoquant 2.5 software.

### 4.8. Statistical Analysis

All quantitative data were expressed as the mean ± SD. Excel 2016 software was used for statistical analysis by the *t-*test. The statistical data were evaluated using a two-sided test (* *p* < 0.05 and ** *p* < 0.01 were considered statistically significant).

## 5. Conclusions

In this work, we successfully developed two novel molecular probes, [^99m^Tc]Tc-CN5FA and [^99m^Tc]Tc-CNPFA, to target FR without further purification. In vitro and in vivo studies demonstrated the high affinity and specificity of these probes for targeting FR. As they can be prepared as a kit formulation, they are promising candidates as tumor imaging agents to target folate receptors.

## Figures and Tables

**Table 1:** Biodistribution of [^99m^Tc]Tc-CN5FA and [^99m^Tc]Tc-CNPFA (mean ± SD for *n* = 3).

	[^99m^Tc]Tc-CN5FA			[^99m^Tc]Tc-CNPFA		
Tissue	0.5 h	2 h	2 h-block	0.5 h	2 h	**2** **h** **-block**
Heart	2.60 ± 0.16	2.29 ± 0.26	0.84 ± 0.05	1.92 ± 0.06	1.30 ± 0.09	0.81 ± 0.12
Liver	13.91 ± 1.27	11.88 ± 1.22	10.22 ± 0.43	15.84 ± 1.19	10.39 ± 0.23	10.88 ± 1.03
Lung	2.88 ± 0.09	2.78 ± 0.57	2.27 ± 0.16	4.01 ± 0.30	2.04 ± 0.06	2.11 ± 0.28
Kidney	47.20 ± 2.99	51.92 ± 3.10	35.40 ± 4.08	81.30 ± 4.69	80.25 ± 1.23	37.60 ± 2.46
Spleen	3.07 ± 0.03	3.08 ± 0.42	4.52 ± 0.38	3.65 ± 0.72	2.85 ± 0.26	3.30 ± 0.66
Stomach	2.17 ± 0.70	2.00 ± 0.33	1.70 ± 0.16	6.67 ± 0.73	3.65 ± 0.75	2.18 ± 0.22
Bone	1.03 ± 0.09	0.87 ± 0.16	1.02 ± 0.18	1.77 ± 0.06	0.79 ± 0.11	1.02 ± 0.10
Muscle	1.72 ± 0.10	1.27 ± 0.03	0.61 ± 0.10	1.48 ± 0.07	0.84 ± 0.04	0.51 ± 0.09
Intestines	2.35 ± 0.79	1.38 ± 0.15	1.39 ± 0.15	4.98 ± 1.57	1.60 ± 0.24	1.70 ± 0.22
Blood	2.36 ± 0.10	1.08 ± 0.03	1.05 ± 0.08	4.08 ± 0.54	1.46 ± 0.03	1.97 ± 0.34
Tumor	3.03 ± 0.10	2.42 ± 0.44	1.53 ± 0.10	4.18 ± 0.36	2.53 ± 0.12	1.62 ± 0.08
Thyroid (%ID)	0.05 ± 0.00	0.03 ± 0.01	0.03 ± 0.00	0.08 ± 0.02	0.06 ± 0.01	0.05 ± 0.01
T/M ^1^	1.76	1.91		2.82	3.01	
T/B ^2^	1.28	2.81		1.02	1.73	

^1^ T/M = tumor/muscle ratio; ^2^ T/B = tumor/blood ratio.

## Data Availability

The data presented in this study are available on request from the corresponding author.

## Sample Availability

Samples of the compounds are available from the authors upon reasonable request.
